# In the Interest of Peace

**Published:** 1886-11

**Authors:** 


### IN THE INTEREST OF PEACE.

"ANGRY WORDS STIR UP STRIFE."

By reference to our correspondence department, it will be seen that the "East Line Medical Association" essay to defend Dr. Becton (from an imaginary assault) by *attacking us!*

The zeal of the doctor's friends is to be commended,—up to a certain point; but when two of them exceed him, and stop their own JONRNAL, injury is inflicted only on themselves.

When, in July, we said "the majority of the members of the State Medical Association deprecate the introduction of a religious element into thc Presidential addresses," and pointed out certain language of Drs. Brown and Becton which we thought was "untimely and out of place," we were sincere in the desire to preserve harmony in the association, feeling assured that such language would provoke retorts from members, who, holding adverse views, had been denounced as "misguided",—and thus create feeling and discord which might even break up the association.

We have since pointed out that the "sentiment", which, we said, was not "endorsed by the majority of the members," was the assertion by the President that "the great body of American physicians KNOW that the doctrine of Darwin and Huxley are as withering, blighting as the winter's frost; that they "freeze," "damn." etc., etc., etc. We said specifically, "THIS DECLARATION commits the association to sentiments which are foreign to a majority of the members;" and it was the *sentiment on* THIS SUBJECT, which "THE JOURNAL, to some extent, at least," claimed to reflect. We denied that the profession KNOW any such thing.

The meaning should be clear to an unprejudiced person; yet the language, having a specific reference to that quotation from Dr. Becton's address, wherein HE most unmistakable "assumes to represent the medical fraternity in matters of Theology," is construed by the "East Line Medical Association" into an "assumption" of the kind on our part, and called "audacious and impertinent." As our remarks can only be distorted into such construction, while the language of the President's address is scarcely susceptible of any other, it will be seen that the zeal of the doctor's friends leads them, seemingly, into the absurdity of lashing him, over our shoulders. It does make a difference whose ox is gored. That which is essayed to be defended in a President, *would be* "audacious" in an editor. The "assumption" has been stigmatized by the "East Line Association," and must rest where it properly belongs,—not with us.

Now, the provocation is strong, to "answer back,"—to have the last word. The propensity to do so is one of the most persistent manifestations of the "old Adam" in man's nature, and we plead guilty to our share. It will be seen, by reading our letter to Dr. Garnett, that it *was* our intention to defend ourself from this unwarranted attack by the "East Line Medical Association," by publishing letters from prominent members, wherein the position and language of THE JOURNAL are strongly endorsed,—and that to which we only mildly protested,—denounced in language which would inevitably have lead to strife, and created, perhaps, a fatal split in the association. But we have thought better of it; and having the approval of our own conscience, and being confident of that of all right-thinking and unbiassed members who prefer peace to strife, (whatever may be said of our action by others), we will let the "resolutions" pass; let the "East Line" have the last word ;—*decline* the tendered affront, and bearing the injustice as well as we may, we will have nothing more to say on the subject. This is the proper, the manly course, and will be appreciated.

And rigbt here—lest we may be thought inimical to the gentlemen, on whose accouut all this fuss has been made, we will say that no one in Texas admires and respects them, as gentlemen and physicians, more than we do. It was an *error of judgment* to declare *what* the profession believe or do not believe on the subject of religion or evolution, in an address before the State Medical Asssociation. It was dangerous to its harmony, and foreign to its objects,—and deprecated by the majority of the members, a position which we still maintain.

Our labors in behalf of medicine in Texas are on record and speak for themselves. We have the good of the cause at heart, and will not permit ourselves to be irritated or provoked into doing that which would bring about just what we have sought to avoid; and we distinctly declare that our action in thus finally dismissing the subject (which ought never to have been mooted), is the best evidence of our sincerity, and of our devotion to the cause of honorable medicine in Texas.

